# Within-Day Variability in Negative Affect Moderates Cue Responsiveness in High-Calorie Snacking

**DOI:** 10.3389/fpsyg.2020.590497

**Published:** 2021-01-07

**Authors:** Thalia Papadakis, Stuart G. Ferguson, Benjamin Schüz

**Affiliations:** ^1^College of Health & Medicine, University of Tasmania, Hobart, TAS, Australia; ^2^Institute of Public Health and Nursing Research, University of Bremen, Bremen, Germany

**Keywords:** snacking, ecological momentary assessment, food choices, negative affect, affect variability

## Abstract

**Background:**

Many discretionary foods (“snacks”) contribute both to individual health risks and to global issues, in particular through high carbon footprints and water scarcity. Snacking is influenced by the presence of snacking cues such as food availability, observing others eating, and negative affect. However, less is known about the mechanisms underlying the effects of negative affect. This study examines whether the individual odds of consuming high-calorie snacks as a consequence to being exposed to known snacking cues were moderated by experiencing (i) higher or lower total negative affect per day or (ii) higher or lower negative affect variability per day.

**Methods:**

Secondary analysis of an ecological momentary assessment study of 60 participants over 14 days with food logs and randomly timed assessments of known snacking cues. High total daily negative affect levels (daily within-participant means) and negative affect variability (daily within-participant SDs) were examined as moderators to predict high-calorie snacking in three-level hierarchical random effects logistic regressions.

**Results:**

Consistent with previous studies, the odds of snacking increased when food was available (OR = 5.05, 95% CI 3.32, 7.66), when others were eating (OR = 5.11, 95% CI = 3.73, 6.99), and when participants experienced more negative affect (OR = 1.02, 95% CI = 1.01, 1.03). Associations for food availability (OR = 0.92, 95% CI 0.86, 0.99) and others eating (OR = 0.95, 95% CI 0.91, 0.99) were significantly moderated by negative affect variability such that associations between cues and high-calorie snacking were weaker on days with higher negative affect variability, but not negative affect levels.

**Conclusion:**

The relationship between cues to high-calorie snacking and snacking behavior varies with variability in negative affect, suggesting a complex relationship between affect and high-calorie snacking. Clearer conceptualizations on the relation between affect and eating are needed.

## Introduction

Snacks (i.e., discretionary food choices) are defined as foods that are consumed outside of main meals (Hess et al., 2016). Snacks are key contributors to our overall energy intake, contributing to approximately 24% of an individual’s daily energy intake in the United States (Piernas and Popkin, 2009) and approximately 35% in Australia, the context of the current research (Australian Bureau of Statistics (ABS), 2014). Given that snacking is associated with both high caloric intake and increased consumption, snacking has been linked to greater risk of energy imbalance and weight gain (Hall et al., 2011). Further, discretionary foods contribute substantially to overall food-related greenhouse gas emissions (GHGE). For example almost 30% of food-related GHGE in Australia result from discretionary foods, with an even higher contribution in individual diets high in snacks (Hendrie et al., 2016). Similar patterns have emerged in the United States (Chapa et al., 2020) and elsewhere (e.g., Mehlig et al., 2020). The production of high-energy discretionary foods also consumes substantial amounts of water, and the contribution of discretionary foods to water scarcity has been estimated around 35% (Riddout et al., 2019). Therefore, it is vital to further our understanding of the factors that influence snacking, in order to both mitigate the negative effects of snacking on health and the overall environment, and to promote research aimed at changing obesity related eating behavior.

Early theories of eating behavior (including snacking), assumed the key determinant of eating to be energy depletion, whereby hunger was believed to be predominately driven by a physiological lack of food and a need to restore energy imbalance (Woods et al., 2000). More recent theories (Stroebe et al., 2013), however, posit that eating behavior, in particular snacking, is largely guided by exposure to food-related eating cues or stimuli. Broadly, such theories propose that individuals automatically respond to food-related cues that they encounter in their day-to-day lives and misinterpret their response to such cues as a sign of biological hunger, triggering food consumption.

The relationship between food cues and eating behavior has been explored both under controlled laboratory conditions (Herman et al., 2003; McFerran et al., 2009; Cruwys et al., 2015) and in the real-world (Elliston et al., 2017; Schüz et al., 2018). Social cues, such as observing others eating, have been associated with increased food consumption, influencing both the type and quantity of food consumed (Herman et al., 2003; Cruwys et al., 2015; Herman, 2015). The association between food availability and eating has also been investigated. For example, geographical areas with a higher density of fast-food outlets are associated with increased fast-food consumption (Lucan and Mitra, 2012). Conversely, areas with a high density of fruit and vegetable outlets and supermarkets, are associated with higher fruit and vegetable consumption (Morland et al., 2006; Bodor et al., 2008; Lucan and Mitra, 2012). Recent studies examining snacking behavior in everyday contexts indicate that both social cues (e.g., being in the presence of someone else eating) and having food available significantly increases the likelihood of snacking (Schüz et al., 2015a; Elliston et al., 2017).

### Negative Affect and Eating Behavior

Food-related or snacking cues may also be internal, such as different emotional states that may trigger hunger or prompt someone to eat (Lutter and Nestler, 2009). Most research on affective states has focused on negative affect as a key precipitant to snacking behavior. For example, negative affect has been linked with increased appetite and unhealthy snack choices (Cleobury and Tapper, 2014). Further, negative emotions such as anger, fear, and sadness have been associated with increased impulsive eating and the consumption of unhealthy foods (Macht, 2008). Other research has suggested that negative affect leads to snacking as eating might serve the purpose of down regulating negative emotions (i.e., “comfort eating”) in some individuals (Macht et al., 2005). Finally, some research indicates that negative affect may influence snacking when it is used as a coping strategy to distract oneself from stress (masking hypothesis; Polivy and Herman, 1999). Findings from recent studies examining snacking in everyday contexts indicate that higher levels of negative affect are associated with an increased likelihood of having a snack (Elliston et al., 2017).

While the association between negative affect and snacking has been well documented, it is currently unclear whether negative affect has a direct effect on snacking or, instead, acts via mediators. For example, some research has suggested that negative affect might perhaps impair cognitive control over eating, leading to increased snack consumption (Macht, 2008). This idea has been posed by two potential theoretical explanations. One explanation is that experiencing high levels of negative affect disinhibits dietary restraint, leading to increased snacking (Herman and Polivy, 1984). According to this view, negative affect is thought to pose a more urgent and current concern to the individual than regulating their food consumption. Specifically, there is a greater demand on the individual to manage this more urgent stressor (the negative emotion) than to focus on their diet. Consequently, cognitive control over eating is impaired, leading to greater snacking. For our study, this means that total daily negative affect could act as a moderator of cue effects on snacking, with e.g., higher total daily negative affect being associated with stronger effects of cues on snacking (as a result of impaired control).

### Negative Affect Variability and Eating Behavior

An alternative explanation has been proposed by self-regulation theories, which pose that individuals’ self-regulatory capacities are a limited resource, that are depleted when people attempt to control their emotions, thoughts and behavior (Martin Ginis and Bray, 2010). Specific research on emotion regulation indicates that attempting to change/control momentary negative affect reduces blood glucose levels, which consequently reduces performance on subsequent self-regulation tasks (Muraven et al., 1998). In this study, participants were asked to engage in an emotion self-regulation task (changing their emotions whilst watching an upsetting movie), and then to engage in a subsequent self-regulation task based on physical exertion and stamina (to continuously squeeze a handgrip). Results have been interpreted as indicating that trying to control/alter one’s emotional state leads to a reduced capacity to self-regulate in another area.

If self-regulatory capacity is a limited resource that is depleted when people attempt to regulate their emotions, subsequent self-regulation of behavior might also be impaired (Cameron and Overall, 2018). For example, research demonstrated that when participants—restrained eaters, or “dieters”—had to cope with negative emotions, their ability to control their eating was inhibited, leading to higher consumption of high caloric snack foods (Boon et al., 2002). Therefore, when people’s self-regulatory resources have already been depleted (through regulating their negative affective states), they may lack self-regulatory resources and may be more vulnerable to eating in response to cues. In other words, on days when individuals experience their emotions to be more varying, their resources to self-regulate eating behavior might be depleted and they accordingly would be more susceptible to snacking cues and eat more. Previous research in other health behaviors however suggest a heterogeneous picture – higher variability in affect was both related to higher levels of health-promoting behaviors (e.g., diabetes self-care behavior; Wagner et al., 2017) and lower levels of health-promoting behaviors (e.g., physical activity; Maher et al., 2019). This suggests that more research on the role of variability in affect in health behaviors is needed.

In sum, there are two theoretically plausible avenues that outline how negative affect might moderate the effects of food cues on snacking within participants: Higher total daily levels of negative affect might impair cognitive control over eating by posing a more urgent demand, or, secondly, higher daily variability in negative affect could deplete self-regulatory resources, making participants more susceptible to momentary eating cues. As in particular the intake of high-calorie and low-fiber foods has been associated with adverse health outcomes (e.g., Saklayen, 2018) and adverse environmental outcomes such as high GHGE (Hendrie et al., 2016) and water scarcity (Riddout et al., 2019), this research focuses on the discretionary intake (snacking) of high-calorie foods.

### Study Aims

The current study investigates the role of daily negative affect *levels* and daily negative affect *variability* as potential moderating variables of the relationship between internal and external food-related cues (food availability, observing others eating and momentary negative affect) and high-calorie snacking in an everyday setting using Ecological Momentary Assessment methods (EMA; Shiffman et al., 2008). In addition to the hypothesis that internal and external cues would be associated with higher odds of snacking (H1), we tested two competing hypotheses, specifically: that the presence of known snacking cues, in particular food availability, observing others eating and negative affect will be associated with increased odds of snacking, and that these effects will be moderated by total daily negative affect *levels* (H2); and, that the presence of known snacking cues, in particular food availability, observing others eating and negative affect will be associated with increased odds of snacking, that these effects will be moderated by negative affect *variability* (H3).

## Materials and Methods

### Overview

Using EMA (Shiffman et al., 2008) allowed us to identify the presence and intensity of internal and external cues in real world settings and in near real time. Further, EMA allowed us to examine momentary within-person variability and fluctuation in negative affective states. The present study used data from a previously published study (Schüz et al., 2018).

### Participants

This study was a secondary analysis of a previously published study (Schüz et al., 2018) which examined the relationship between momentary social norms and dietary behaviors. Participants were recruited for this study, via newspaper, radio and online media release. To be eligible to participate, individuals were required to be >18 years of age, have a Body Mass Index (BMI) between 18 and 40 kg/m^2^ (i.e., within the normal-to-obese BMI range), not being on a diet, and have no history of an eating disorder.

### Procedure

Data for this study was collected between April and August 2016. The protocol for data assessment followed those outlined in previous published research (Schüz et al., 2015b; Elliston et al., 2017), and was approved by the Tasmanian Social Science Human Research Ethics Committee (Reference No. H0015647). Initially, interested participants contacted the researchers via web form. After establishing eligibility through telephone screening, participants were booked in for the first appointment (∼30 min in duration), during which they provided informed consent, completed baseline measures, and received initial training with the EMA devices (LG P500 smartphones stripped of all phone functions) running the customized EMA software HBART.^1^

Briefly, participants then completed 14 days of EMA field assessment, in which they were instructed to log every time they consumed food or drinks. After 2–3 days, they attended the lab to receive additional training (if necessary) and assess protocol compliance. Food reports were assessed in two stages: Firstly, participants logged all the food and drink they consumed. Secondly, a random subsample (60% to minimize participant burden; Schüz et al., 2013) of these food assessments were followed by assessment of the presence of social, environmental and internal cues to eating. Participants also received randomly-timed prompts over the course of the day (approximately five/day), which repeated the assessment of social, environmental and internal cues to eating (see section “Measurement”). This allowed for the comparison of the presence and strength of food related cues during eating and non-eating assessments. Each assessment was time and date stamped. Participants were instructed to turn the device to “suspend mode” whenever they were in circumstances where they would not be able to answer random prompts (such as when driving). Further, participants completed a brief evening report at the end of each day (for future studies). On conclusion of the monitoring period, participants retuned their EMA devices, were debriefed, and received $50 reimbursement.

### Measurement Instruments

*Food reports* were assessed in two steps. Firstly, participants reported whether they were eating a meal or snack, and secondly identified what kind of food they were eating based on the Dietary Targets Monitor (DTM; Lean et al., 2003). For example, if participants selected “Enter snack report” on the study smartphone, they were asked to “Please indicate which type of food you want to report” with a selection of food groups based on the DTM, e.g., “fruit and vegetable”, “cheese”, “sweets or chocolate”, “cake, scone, sweet pies, danish”, “biscuits”, “ice cream”, etc. Snack reports were then differentiated as either “low calorie snacks” or “high calorie snacks” based on their estimated energy and saturated fat content. For example, fruit and vegetables were classified as low caloric snacks, while sweets and chocolate, chips, ice-cream, cakes/scones/pastries, crisps/savory snacks, and biscuits were classified as high caloric snacks. This study focused solely on high caloric snack intake, given its known association with negative health outcomes (Hall et al., 2011).

*Social cues* were assessed during both food reports and non-eating assessments by asking participants “When you decided to eat, were there people eating?” Responses were qualitative and required answering a single option from: “no,” “yes in my view,” or “yes in my group.” For analysis, responses were dichotomized to yes/no.

*Food Availability* was assessed during both eating events and randomly timed non-eating assessments. Participants were asked what food was available at the time they decided to eat. Responses were qualitative and required participants to check boxes of available food types.

*Affect* was assessed by asking participants to rate their mood at the time they decided to eat across 10 affect descriptors: alert; angry; bored; calm; focused; happy; irritable; stressed; restless; sad; overall mood; and energy level. Descriptors were assessed on a 0–100-point visual analog scale, whereby participants moved a pointer to indicate their response. A maximum likelihood factor analysis with robust standard errors taking into account the hierarchical data structure (multiple measures nested under participants) confirmed a two-factor structure. Responses were then summarized into a positive affect score (using the mean scores for alert, calm, focused, happy, energy) and negative affect (using the mean scores of angry, bored, irritable, stressed, restless, sad).

*Total daily negative affect* was operationalized as the mean of negative affect from all eating and non-eating assessments experienced by a participant during one day (hypothesis 2), with higher scores indicating higher average negative affect on this day.

*Negative affect variability* was operationalized as the within-day and within-person standard deviation of negative affect scores from all eating and non-eating assessments (hypothesis 3). This indicates the degree to which participants’ negative affect scores during any one day deviated from their daily mean, with higher scores indicating days with greater negative affect variability.

### Data Preparation

On average, participants completed 14.57 (SD = 2.41) days of field monitoring. Following the exclusion of days with poor compliance (<50% of random prompts responded to), out of 904 days of participant observations, 776 (85.4%) days of participant observation were available for analysis. In the resulting data set, participants responded to 2,058 of 2,374 (86.69%) non-eating assessments issued, an average of 2.87 (SD = 1.28) random assessments per day. Participants reported consuming 0.96 (SD = 1.26) high-calorie snacks per day.

### Analysis

Due to the hierarchical structure of EMA data, in which multiple daily assessments of food reports and randomly timed reports are nested within both days of the study and within participants, a three-level multilevel analysis with cross level analysis was used to control for the non-independence of observation. The R package lme4 (Bates et al., 2015) was used to obtain estimates of odds ratios and fixed and random effects in the multilevel analysis, and sjPlot (Lüdecke, 2019) was used to graph interactions. Descriptive analyses were conducted using SPSS.

The analyses for our main research questions were conducted through multiple steps. First, we fitted a series of separate multilevel logistic regression analyses per predictor to test hypothesis 1 and to replicate previous findings of positive associations between known momentary internal and external cues (food availability, others eating, momentary negative affect) and snacking. In these models, for each report, the odds that this report was a random prompt (coded 0) or a snack report (coded 1) was regressed on the cues separately in three-level hierarchical linear models (as reports are nested within days, and days are nested within participants).

Next, we tested the second hypothesis, namely that total daily negative affect levels moderated the effects of these known cues on snack reports [Table 2, Model a) for others eating, model b) for food availability, and model c) for negative affect]. To do so, we introduced person-level centered daily means of negative affect as moderators of the known cues and direct predictors of snacking (cross-level interactions) into the hierarchical logistic regression analyses. To test the third hypothesis, similar models were fitted that introduced person-level centered NA variability (daily within-participant SDs) in the multilevel logistic regression. Day-level predictors (NA level and NA SD) were person-mean-centerd in order to indicate days on which participants experienced higher (or lower) levels and variability of NA than on average.

All analyses included study day (within-participants) as covariate to control for time effects in the study. The odds ratio indicates how much more or less likely it is that any report is a snack report compared to being a non-eating assessment, if the specific covariate increases by one unit. For the categorical covariates (food availability and other eating), the odds ratio indicates the likelihood of snacking if the covariates are present vs. absent. In the case of negative affect, the odds ratio indicates the likelihood of snacking with a one-unit increase in negative affect (note that these effects appear small, as they indicate the increase in odds if negative affect increases by 1 unit on a scale from 0 to 100).

## Results

A total sample of 61 adults was assessed, and 60 (98.36%) of this sample provided data on a sufficient number of days (>2) to allowed inclusion in these analyses. Of these subset (*n* = 60), 41 (69%) were women. Participants were aged between 18 and 64 years (*M* = 32.37, SD = 12.96) and had an average BMI of 25.04 kg/m^2^. Most participants (*n* = 55, 91.7%) were of Caucasian origin. The majority (n = 33, 55%) had completed some university education, followed by completing high school (*n* = 20, 33,3%) or vocational training (*n* = 7, 11.2%). Descriptives for internal and external cues (% present in measurement occasions) as well as negative affect levels and variability can be found in Table 1.

**TABLE 1:** Means and standard deviations (sample-level) and% present of internal/external cues and moderators (negative affect levels and variability).

**Variable**	**Mean**	**SD**	**% present during measurement occasion**
**Internal cue**			
Momentary negative affect	18.30	17.81	
**External cues**			
Others eating			24.75
Food available			80.66
**Moderators**			
Negative affect level	18.30	15.15	
Negative affect variability	8.22	4.08

Hypothesis 1: Are known cues (available food, others eating and momentary negative affect) associated with snacking?

As hypothesized, all known cues were associated with an increased likelihood of snacking. Results indicated that the presence of others eating significantly increased the odds of a measurement occasion being a snack (OR = 5.11, 95% CI = 3.73, 6.99) when compared to non-eating assessments. Similarly, food availability significantly increased the odds of a measurement occasion being a snack (OR = 5.05, 95% CI = 3.32, 7.66) when compared to non-eating assessments. The intensity of momentary negative affect significantly increased the odds of a measurement occasion being a snack (OR = 1.02, 95% CI = 1.01, 1.03) when compared to non-eating assessments (note that momentary negative affect was assessed on a 0–100 scale, thus small increases in odds with one unit increase in momentary negative affect).

Hypothesis 2: Are the associations between known cues (available food, others eating and momentary negative affect) and snacking moderated by total daily negative affect *levels*?

To test hypothesis 2, daily within-person per-day average negative affect levels were examined as a moderator of the relationship between known internal and external cues (food availability, observing others eating, and negative affect) and snacking. Results (see Table 2) indicated that total daily negative affect did not moderate the relationship between others eating and snacking (OR = 0.97, 95% CI = 0.93, 1.01), food availability and snacking (OR = 0.97, 95% CI = 0.93, 1.02), or momentary negative affect and snacking (OR = 1.00 95% CI (0.99, 1.00).

**TABLE 2:** Summary of 3 three-level multilevel analyses: snacking regressed on internal and external cues using average negative affect (NA) per day as a moderator.

	**Odds Ratios (95% CIs) of External and Internal Cues**		
	**(a) Availability of Food**	**(b) Others Eating**	**(c) Negative Affect**
**Fixed Effects (Occasion level)**			
Intercept	0.23 (0.17, 0.30)***	0.25 (0.13, 0.32)***	0.24 (0.20, 0.30)***
Cue (main effect)	5.38 (3.44, 8.41)***	4.96 (3.49, 7.06)***	1.02 (1.01, 1.04)***
**Fixed Effects (Day Level)**			
Day in study	0.97 (0.94,0.99)*	0.97 (0.94,0.99)*	0.98 (0.95,1.00)
NA Level * Intercept	1.00 (0.99, 1.02)	1.00 (0.99, 1.01)	1.00 (0.99, 1.01)
NA Level * Slope Cue	0.97 (0.93, 1.02)	0.97 (0.93, 1.01)	1.00 (0.99, 1.00)
**Random Effects**	**Variance Component (SD)**	**Variance Component (SD)**	**Variance Component (SD)**
σ2 ntercept Level-1	0.29 (0.54)	0.35 (0.58)	0.00 (0.00)
σ2 Intercept Level-1/2	0.00 (0.00)	0.00 (0.00)	0.00 (0.00)

*p < 0.05, ***p = 0.001. NA, Negative Affect; SD, Standard Deviation.

Hypothesis 3: Are the associations between known cues (available food, others eating and negative affect) and snacking moderated by negative affect *variability*?

**FIGURE 1:**
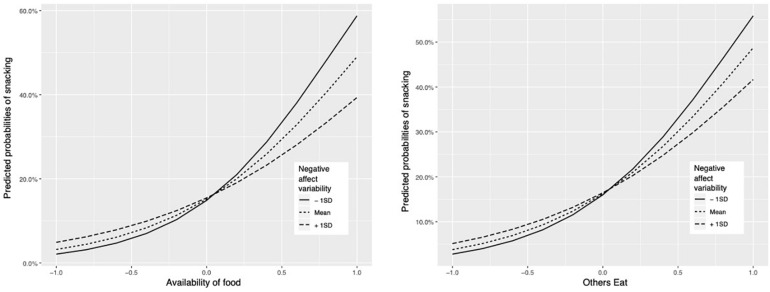
Predicted probabilities of a measurement occasion being a snack report by external cue (others eating/availability of food) and negative affect variability (centered predictors).

To test hypothesis 3 | daily within-person per-day negative affect variability was examined as a moderator of the relationship between known internal and external cues and snacking. Results (see Table 3 and Figure 1) indicated that negative affect variability was a significant moderator of the relationship between food availability and snacking (OR = 0.92, 95% CI = 0.86, 0.99) and observing others eating and snacking (OR = 0.95, 95% CI = 0.91, 0.99). Negative affect variability did not moderate the relationship between and momentary negative affect and snacking (OR = 1.00, 95% CI = 0.99, 1.00). This means that negative affect *variability* moderated the relationship between known external cues such that these relationships were weaker on days with more variability in negative affect, but not internal (momentary negative affect). Figure 1 shows the predicted probabilities of a measurement occasion being a snack report based on the presence of external cues (availability and others eating) and the variability of negative affect per day (centerd predictors, thus mean = 0).

**TABLE 3:** Summary of three-level multilevel analyses: snacking regressed on internal and external cues using negative affect variability (day-level) as a moderator.

	**Cues Odds Ratios (95% Cis)**		
	**Availability of Food**	**Others Eating**	**Negative Affect**
**Fixed Effects (Occasion level)**			
Intercept	0.23 (0.18, 0.30)***	0.14 (0.09, 0.20)***	0.25 (0.20, 0.31)***
Cue (main effect)	5.35 (3.38, 8.46)***	6.26 (4.10, 9.56)***	1.02 (1.01, 1.04)**
**Fixed Effects (Day Level)**			
Day in study	0.97 (0.94,0.99)*	0.98 (0.95,1.00)	0.98 (0.95,1.00)
NA SD * Intercept	1.00 (0.98, 1.03)	1.02 (0.99, 1.04)	0.99 (0.97, 1.02)
NA SD * Slope Cue (moderation)	0.92 (0.86, 0.99)*	0.95 (0.91, 0.99)*	1.00 (0.99, 1.00)
**Random Effects**	**Variance Component (SD)**	**Variance Component (SD)**	**Variance Component (SD)**
σ2 Intercept Level-1	0.37 (0.60)	0.37 (0.61)	0.00 (0.00)
σ2 Intercept Level-1/2	0.00 (0.00)	0.00 (0.00)	0.00 (0.00)

*p < 0.05, ***p = 0.001. NA, Negative Affect; SD, Standard Deviation.

## Discussion

In a secondary analysis of a previous data set, this study examined the influence of negative affect variability and negative affect levels on cue susceptibility in high-calorie snacking behavior in everyday settings. The consumption of high-calorie snacks has been linked to negative outcomes on individual health (Saklayen, 2018), while the production of such snacks has negative effects on GHGE (Hendrie et al., 2016), and water scarcity (Riddout et al., 2019). Consistent with previous research, participants were more likely to consume a high-calorie snack when exposed to known internal and external snacking cues such as availability of food and others snacking (external cues) and negative affect (internal cue). This finding supports previous research that suggests that snacking is highly influenced by an individual’s situation and context (Lowe and Butryn, 2007) and corroborates a number of studies that show that internal and external snacking cues increase the likelihood of snacking (Schüz et al., 2015a; Elliston et al., 2017). More importantly though, these effects were moderated by daily negative affect variability (Table 3), but not total daily negative affect levels (Table 2).

This means that greater negative affect variability led to a lower susceptibility to external snacking cues. This finding is in contrary to our hypothesis that susceptibility is increased on days with more fluctuation due to potential self-control depletion effects (Muraven et al., 1998), but would suggest the opposite – greater fluctuations of negative affect within days and within participants are associated with smaller effects of known snacking cues. There are some potential explanations for this unexpected finding, in particular related to attention effects. Greater affect variation has been associated with fluctuations in attention and working memory (Brose et al., 2012), thus participants might have paid less attention to snacking cues on days with larger fluctuations in negative affect. Variability in negative affect did not affect the odds of snacking directly, however. A previous studies report heterogeneous findings for the relationship between affect variability and health behavior (e.g., higher levels of diabetes self-care behaviors with higher fluctuations of positive affect; Wagner et al., 2017 but also lower levels of physical activity with higher fluctuations in affect; Maher et al., 2019), our results add to this literature that in addition to main effects of variability on health behavior, potential moderating effects of affect variability on the relationship between behavioral cues and behavior need to be considered.

Importantly, total daily negative affect levels did not moderate the associations between known cues and high-calorie snacking. Therefore, experiencing high levels of negative affect on any given day did not increase participants’ susceptibility to snacking cues. Theoretically, this finding suggests that negative affect levels and negative affect variability have slightly different implications for snacking. Whilst high negative affect levels are directly associated with increased odds of high-calorie snacking (as an internal cue), negative affect variability appears to modify the susceptibility to a range of known cues. This suggests t that negative affect not only serves as a cue to high-calorie snacking *per se*, but the experience of greater variability of negative affect could affect attentional processes related to cue detection.

At the same time, the observed fluctuations in negative affect could also be the result of individual affect regulation processes – if individuals experience high levels of momentary negative affect and have the resources and ability to down-regulate this experience to lower levels, this would result in overall higher fluctuations in negative affect per day. Instead of indicating lower self-regulatory resources however (Muraven et al., 1998), it could be indicative of better affect regulation skills, which in turn have been associated with higher levels of health-protective behaviors in general (DeSteno et al., 2013), dietary behaviors in particular (e.g., Isasi et al., 2013), and smaller effects of food cues on dietary behaviors (e.g., Kerin et al., 2018). However, as our study did not assess emotional regulation but instead examined naturally occurring variability in affect, this possible pathway could neither be confirmed nor rejected.

Given that existing reviews on the role of affect in dietary behaviors (Macht, 2008) are mainly concerned with intensity (level) of affect as determinant of eating, more conceptual and theoretical work on the relationship between affect variability and eating is needed. Because there are fluctuations in affect over the course of days (standard deviations of negative affect in our study ranged from 0 to 40 on a 0–100 scale), both within- and between-day fluctuations in affect need to be considered. The evidence from this study can only be considered initial, as both the relatively small sample size and exploratory nature of the study as a secondary analysis limit the implications of the findings. Future research may focus on manipulating negative affect variability in controlled laboratory settings, in order to examine individuals’ reactivity to snacking cues.

More broadly, findings from the current study suggest a greater need for interventions that target and address food cues, given that all three cues included in this study (food availability, observing others eating, and negative affect) were associated with increased odds of high-calorie snacking. Addressing these food cues could therefore be an important focus of measures to reduce snack food consumption, given the current obesogenic environment whereby individuals are continuously exposed to/bombarded by snacking cues. Both in terms of reducing the health risks associated with excess energy and fat consumption (Saklayen, 2018) as well as the environmental impact of discretionary foods (Hendrie et al., 2016; Riddout et al., 2019; Chapa et al., 2020; Mehlig et al., 2020), effective measures are needed. For example, recent research suggests that attentional bias modification training can help people to withstand snacking cues, such as television advertisements for chocolate (Kemps et al., 2018). At the same time, changes in dietary patterns toward more sustainable diets correspond with lower carbon emissions (Mehlig et al., 2020). However, further research is required to ascertain whether these effects can be generalized to other known snacking cues.

A key strength of this study was that it was the first to examine the effects of negative affect levels and negative affect variability on cue susceptibility using EMA (Shiffman et al., 2008). EMA allows for the real-time study of individuals in their everyday eating environments, capturing the experience of moods, behavior and events that occur prior to eating.

Despite this strength, there are some important limitations to consider when interpreting the results of the present study. First, participants’ high-calorie snack intake could not be verified as EMA relies on self-reports of eating behavior. Second, our assessment of eating via food logs was limited to a brief questionnaire based on a dietary targets monitor (Lean et al., 2003). This measure is limiting as it does not assess the amount of food consumed by a participant, which is an important contributing factor. However, using this measure reduced assessment and time burden on participants and likely increased their compliance to the EMA procedure. Given the relatively small sample size, and the exploratory nature of this study as a secondary analysis, findings from the present study require replication to ensure reliability.

In conclusion, this study provides initial evidence that daily within-participant negative affect variability but not negative affect level moderates cue susceptibility to external snacking cues. This suggests the need for more conceptual work on the relationship between variability in affect measures and dietary behaviors. Nevertheless, understanding the links between affect and discretionary food choices is an important prerequisite for the development of effective measures to reduce the negative health and environmental impact from excess consumption of high-calorie snacks.

## Data Availability Statement

The raw data supporting the conclusions of this article is available at https://rdp.utas.edu.au/metadata/bae6e09e-ab11-4d01-8b1a-9bbaf0e6ff73. Analysis code will be made available upon request.

## Ethics Statement

The studies involving human participants were reviewed and approved by University of Tasmania Human Research Ethics Committee. The patients/participants provided their written informed consent to participate in this study.

## Author Contributions

TP conducted analyses, wrote the manuscript, and contributed to conceptualization of the study. SF contributed to the conceptualization of the study and writing of the manuscript. BS conceived the study, conceptualized the analyses, and contributed to writing the manuscript. All authors contributed to the article and approved the submitted version.

## Conflict of Interest

The authors declare that the research was conducted in the absence of any commercial or financial relationships that could be construed as a potential conflict of interest.

## Footnotes

1 https://www.utas.edu.au/health/research/groups/tasmanian-school-of-medicine/behavioural-and-situational-research-group-bsrg/hbart
